# The Effect of Ammonia Inhalants on Mental-Fatigue-Related Force Loss

**DOI:** 10.3390/jfmk10040406

**Published:** 2025-10-18

**Authors:** Matthew J. Barnes, Emma O’Connor, Jason van Zanten

**Affiliations:** School of Sport, Exercise & Nutrition, College of Health, Massey University, Palmerston North 4410, New Zealand; emmac50@gmail.com (E.O.); jasonvanzanten@hotmail.co.nz (J.v.Z.)

**Keywords:** mental fatigue, ammonia inhalants, electromyography, profile of mood states, isometric force

## Abstract

**Objectives:** Ammonia inhalants (AIs) are commonly used in competition with the assumption that they will increase arousal and reduce the detrimental effects of mental fatigue on performance. However, as the effect of AIs on mental fatigue is unclear, this study investigated (1) whether mental-fatigue-related changes in mood states are associated with reductions in maximal lower-body force production and (2) whether AIs reduce any mental-fatigue-induced changes in performance. **Methods:** In a randomized, crossover designed study, nine resistance trained males completed two trials, with and without AIs. Profile of mood states, isometric midthigh pull force, and electromyography were measured before and after completion of a 75 min AX-continuous performance test (AX-CPT). For AI trials, AIs were used prior to post-AX-CPT IMTPs. **Results:** The AX-CPT significantly increased all negative mood subscales, while decreasing vigor (all *p* < 0.05), resulting in an increase in total mood disturbance (pre-AX-CPT: 27.1 ± 3.17 vs. post-AX-CPT: 64.49 ± 4.01; *p* = 0.005). Additionally, compared to baseline, force was reduced immediately (1699 ± 345 vs. 1521 ± 324 N; *p* = 0.009), but not five minutes post-AX-CPT (*p* = 0.328). Electromyography did not change over time, and no differences between treatments were evident for any of the measures. **Conclusions:** Mental fatigue, and related mood disturbance, has the potential to acutely reduce lower-body, maximal force. This finding may have implications for athletes competing in strength sports where mental focus, arousal and maximal force production determine optimal performance. However, AIs offer no benefit to alleviating the detrimental effects of mental fatigue.

## 1. Introduction

Strength sports, such as powerlifting, Olympic weightlifting and strongman require intermittent maximal, or near maximal, muscular efforts over multiple hours, or even days. Along with extreme physiological demands being placed on the athlete, each effort is likely to be associated with repeated psychological stress caused by psyching up and down in order to manipulate arousal and increase subsequent muscular performance [1,2]. Additional psychological demands may occur in preparation for competitions, with changes in tension, anger and depression noted in a recent powerlifting case study [3]. Although it is currently unclear whether the accumulated psychological demands of competition are detrimental to performance in strength sports, powerlifters have previously reported that they are both physically and mentally fatigued towards the end of a competition [4], suggesting that mental fatigue may occur in this setting.

Characterized by tiredness, exhaustion and task disengagement [5], mental fatigue is a psychophysiological state caused by sustained cognitive effort, alone or in combination with physical loading [6]. Prolonged mental exertion leads to increased extracellular adenosine accumulation, particularly in the basal forebrain, which inhibits neuronal activity [7]. This is accompanied by altered dopaminergic signaling in the prefrontal cortex and anterior cingulate cortex [7], and alterations in the functional organization [5] and activity of regions of the brain that regulate motivation, effort, attention, and motor control [7,8].

These changes can have detrimental effects on activities that require sustained cognitive effort, with the level of task complexity appearing to be a key factor [9]. As such, the effects of mental fatigue on athletic performance are greatest for activities that involve prolonged cognitive effort, such as aerobic/endurance exercise [9]. Similarly, due to the associated cognitive load, muscular endurance [10,11], sustained force production [12] and sport-specific psychomotor performance and skill [13,14] are also negatively affected by mental fatigue. Additionally, mood and ratings of perceived exertion during submaximal resistance exercise are also adversely altered [6]. On the other hand, evidence for the effects of mental fatigue on maximal force production is less consistent. For example, mental fatigue has been shown to reduce knee extensor [15], dorsiflexion and handgrip MVIC force [16]. However, conversely, others have reported no change in force during the isometric midthigh pull (IMTP) [12], and dorsiflexion and [17] knee extensor [18] MVICs. Despite a lack of consistent evidence to show that mental fatigue affects maximal force production [13], the findings outlined above suggest that mental fatigue has the potential to impact many aspects of strength sport performance.

In order to combat perceived mental fatigue, and to increase arousal in competition, athletes commonly use stimulants or arousal techniques [4]. Ammonia inhalants (AIs), composed of ammonia carbonate mixed with perfume, are particularly popular among athletes competing in strength sports due to their fast-acting stimulation of the respiratory system. Inhalation of AIs irritate the nerve endings within the nose, nasal cavity, and lungs, triggering an inhalation reflex that may improve alertness and arousal [19]. A survey of 256 International Powerlifting Federation (IPF) athletes found that almost half had used AIs in competition, with 78% of users stating that they thought it improved their performance. Most commonly, athletes reported using AIs prior to the deadlift, the last exercise completed in powerlifting competitions, to mitigate against the fatigue accumulated near the end of a meet [4].

Despite its popularity, the acute use of AIs prior to resistance-based exercise has not been linked to improvements in physical performance (reviewed by Bender & Popkin [19]). However, despite not seeing an improvement in muscular performance with AI use, Campbell et al. [20] reported an 11.5% increase in alertness, based on a visual alertness scale, following inhalation of AIs, compared to a placebo. This finding suggests that AIs may be useful when in a fatigued state, as occurs towards the end of strength sport competitions. However, the effects of AIs have not been investigated in mentally fatigued individuals.

Therefore, the aims of this study were to (1) determine whether expected changes in mood states, caused by completion of the AX-continuous performance test (AX-CPT), are associated with reductions in maximal lower-body force production and (2) whether AIs could mitigate against any mental-fatigue-induced changes in performance. As such, we hypothesized that (1) the AX-CPT would induce mental fatigue and reduce IMTP force, and (2) that AIs would reduce this effect.

## 2. Materials and Methods

### 2.1. Experimental Design

**Figure 1:**
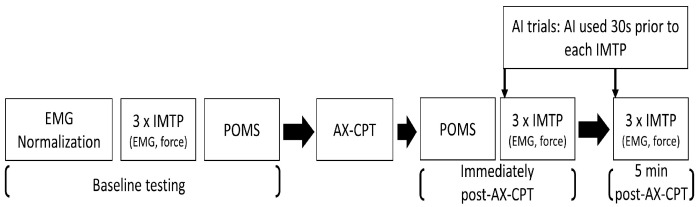
Schematic of the study protocol. Profile of mood states (POMS), isometric midthigh pull force (IMTP) and electromyography (EMG) were measured at baseline, and again after completing the 75 min AX-continuous performance test (AX-CPT) and second POMS questionnaire, and 5 min later. For ammonia inhalant (AI) trials, participants inhaled AIs 30 s before each post-IMTP. This protocol was repeated on two different occasions, under AI and control conditions; at least five days separated the trials, which were performed at the same time of day on each occasion.

This study used a repeated-measures crossover design (two trials (AI vs. control) × three time points (pre-, immediately and five-minutes post-AX-CPT)). Block randomization was implemented in Microsoft^®^ Excel^®^ (version 2205, Microsoft Corporation, Redmond, WA, USA) to ensure that treatments were allocated in a balanced order. Trials were separated by a minimum of five days and were performed at the same time of day to avoid diurnal variation. A study flowchart is presented in Figure 1.

### 2.2. Participants

A priori power analysis, for a repeated measures within factors design, was carried out in G*Power v3.1.9.7 software [21] to identify the number of participants needed to achieve a large change in vigor [6] and fatigue [22] after completion of the AX-CPT. Using Cohen’s *f* = 0.80, power = 0.80, and *α* = 0.05, six participants were needed for this study.

To account for possible dropouts, 10 healthy, resistance-trained males were recruited for the study. The criteria for inclusion were participation in strength training at least three times per week for the previous year; a lack of AI use during that period; an absence of respiratory illness or disease; and the absence of any contraindications to exercise or other procedures involved in the research. None of the participants reported having used AIs before. One participant failed to complete both trials; therefore, analysis was performed on data from nine participants (mean ± standard deviation (*SD*) age 23.3 ± 7.4 years, height 177.9 ± 7.4 cm and weight 82.5 ± 15.7 kg). Prior to inclusion, the participants completed a health screening questionnaire and were notified of all potential risks and benefits associated with the study, before signing an institutionally approved informed consent document. This study was approved by the University Human Ethics Committee (MUHEC14/77), in accordance with the Declaration of Helsinki. Participants were asked to abstain from resistance training, alcohol and stimulants (including caffeine) for at least 24 h prior to each trial, and diet was self-recorded on the first day of testing and replicated for the second trial. Diet records were not analyzed. Participants were familiarized with the IMTP, profile of mood states (POMS) questionnaire, ammonia inhalation, and AX-CPT at least seven days before their first trial.

### 2.3. Procedures

Trials were completed at the same time of day, in the same air-conditioned laboratory with the temperature set to 18 °C to ensure consistency across each trial. Trials started with electromyography (EMG) normalization, during which the participant was seated on a custom-made, straight-backed chair with their hip and knee joints positioned at 90° [23]. An inextensible strap, connected to a load cell (Sensortronics, Convina, CA, USA), was attached to the chair just above the level of the lateral malleolus, and a seatbelt was fixed across the hips to isolate movement to the knee joint and, therefore, the quadriceps muscles. EMG electrodes (Ambu Blue Sensor, Copenhagen, Denmark) were placed on the vastus lateralis of the right leg in accordance with the recommendations of Hermens et al. [24] and a ground electrode was positioned on the right patella. It has previously been demonstrated that the vastus lateralis is highly active during lower limb strength exercise, making this an appropriate sampling location for use during the IMTP [25]. Participants completed three × three second MVICs of the right leg quadriceps muscles by extending at the knee with as much force as possible. Efforts were separated by a 30 s rest. EMG data were collected at 1000 Hz using a PowerLab acquisition system (ADinstruments, Bella Vista, NSW, Australia) and then full wave rectified and processed in Chart for Windows software (V8.1 ADinstruments, Bella Vista, NSW, Australia). EMG activity from a 500 ms sampling window, centered on the peak force obtained during each MVIC, was averaged, and used to represent 100% activity. These data represented the maximal activity level of the vastus lateralis and were used to normalize data collected during the IMTP in that session.

Following EMG normalization, participants completed three maximal IMTPs, separated by one minute rests, on a custom-made lower-body dynamometer composed of a modified barbell and chain attached to a calibrated s-beam load cell (Muller, Meckesheim, Germany) and platform [26]. The load cell was connected to a custom-made amplifier with force output sampled at 1000 Hz with a PowerLab data acquisition system and displayed in Chart for Windows. To ensure consistency between trials, IMTPs were conducted with knee and hip angles of 140° and 120°, respectively, in accordance with Kawamori et al. [27] the position was confirmed prior to each IMTP using a goniometer. The use of weightlifting chalk was optional, but its use (or non-use) had to be consistent for all efforts. For each IMTP, participants were instructed to “pull as hard as possible” until told to stop. Each effort was preceded by a countdown and held for two seconds. The maximum peak force value, and associated EMG data, obtained from the three efforts was used for analysis.

After baseline measures, participants completed a POMS questionnaire to determine their level of TMD. For this, participants ranked 65 words/statements describing feelings grouped into six subscales (tension, depression, anger, vigor, fatigue and confusion) on a five-point scale based on the extent to which they were experiencing each one at the moment of taking the test. Total mood disturbance (TMD) was calculated by summing the scores of all subscales with vigor weighted negatively [28]. Participants then completed a 75 min-long AX-CPT in which sequences of letters requiring a choice of two answers were presented, and a response was required from the participants within 400 ms [29]. There is evidence that the AX-CPT is cognitively demanding, requiring use of the working memory and sustained attention, and it has been associated with significant elevation of the anterior cingulate cortex, an area of the prefrontal cortex that is associated with mental fatigue [22]. The AX-CPT has been used in previous research to demonstrate an association between mental fatigue and perceived exertion during exercise [30] and changes in POMS [31]. The task was completed in a quiet, supervised room to prevent external interference, and participants completed the task with their dominant hand. Following the AX-CPT, participants completed a second POMS questionnaire.

To assess the effects of the mentally fatiguing task on strength performance, three IMTPs were performed immediately and five minutes after completing the second POMS questionnaire, using the protocol described for baseline testing. For ammonia trials, in accordance with Perry et al. [32], a capsule containing 0.33 mL of AI, comprising 35% alcohol and 15% ammonia (Dynarex Corporation, Orangeburg, NY, USA), was crushed 30 s prior to each IMTP to release ammonia fumes. The AI was immediately held under the participant’s nose until a voluntary withdrawal reflex was witnessed.

### 2.4. Statistical Analysis

The statistical analyses were carried out using SPSS Statistics (version 28.0.1.1, IBM Corporation, Armonk, NY, USA). Statistical significance was accepted when *p* ≤ 0.05. The results are presented as the mean ± SD. Dependent variables (peak force, EMG and mood state) were analyzed with a two-way (treatment × time) repeated-measures analysis of variance (ANOVA) to determine the main effects of, and interactions between, treatment (CON vs. AI) and time (baseline and immediately and five minutes after completion of the AX-CPT and second POMS questionnaire). When a significant effect was observed, post hoc analysis with Bonferroni adjustment was carried out. Data were checked for sphericity using the Mauchly method, but no adjustment was necessary. Data were assessed for normal distribution using Shapiro–Wilk tests and Q-Q plots, and for outliers using box plot inspection. The POMS subscales of depression, anger, and confusion were not normally distributed; therefore, a series of Friedman tests were performed to assess differences between treatments and changes over time.

Effect sizes are presented as partial eta square (*ηp*^2^) with 0.01, 0.06, and 0.14 representing small, medium, and large effects, respectively [33]. For pairwise comparisons, Cohen’s *d* and 95% confidence intervals [95% *CI*] were calculated with d = 0.2, 0.5 and 0.8 representing small, medium, and large effect sizes, respectively [33].

## 3. Results

### 3.1. Mood Disturbance

TMD significantly increased over time (*F* (1, 8) = 18.303, *p* = 0.005, *ηp*^2^ = 0.647) from baseline (27.1 ± 3.17) to post-AX-CPT (64.49 ± 4.01), but there was no significant difference between treatments (*F* (1, 8) = 1.832, *p* = 0.213, *ηp*^2^ = 0.186). There were significant increases in the tension (*F* (1, 8) = 5.673, *p* = 0.044, *ηp*^2^ = 0.415), and fatigue (*F* (1, 8) = 18.178, *p* = 0.003, *ηp*^2^ = 0.694) subscales and a significant decrease in the vigor subscale (*F* (1, 8) = 53.379, *p* < 0.001, *ηp*^2^ = 0.870) from baseline to post AX-CPT. Similarly, the results of the Friedman tests showed a change over time for depression (*X*^2^ (1) = 6.250, *p* = 0.012), confusion (*X*^2^ (1) = 7.12, *p* = 0.008), and anger (*X*^2^ (1) = 9.94, *p* = 0.002). There was no significant difference in these measures between treatments (Table 1) and no significant treatment × time interactions were found (all *p* > 0.05).

### 3.2. IMTP Force

Significant, large effects of time were found for both peak IMTP force (*F* (2, 16) = 6.466, *p* = 0.009, *ηp*^2^ = 0.447) and the percentage change in force from baseline (*F* (2, 16) = 6.318, *p* = 0.01, *ηp*^2^ = 0.441 Table 2). Post hoc analysis showed that peak force was significantly lower than baseline (1699 ± 335 N) immediately after completing the AX-CPT and second POMS questionnaire (1521 ± 315 N; *p* = 0.031, *d* = −0.55 [−1.88, 0.78]); however, when measured 5 min later, force was not significantly different to baseline (1605 ± 350 N; *p* = 0.328, *d* = −0.27 [−1.59, 1.04]). Similarly, a greater percentage decrease in force from baseline was seen immediately (–9.61 ± 12.2%; *p* = 0.036, *d* = 1.11 [−2.52, 0.29]), but not 5 min (–5.29 ± 9.58%; *p* = 0.277, *d* = −0.78 [−2.14, 0.56]) post-AX-CPT. There were no treatment effects of AI on IMTP force (*F* (1, 8) = 2.014, *p* = 0.194; *ηp*^2^ = 0.201) and no significant treatment × time interaction was found (*F* (2, 16) = 1.846, *p* = 0.190; *ηp*^2^ = 0.187).

### 3.3. EMG

There were no significant time (F (2, 16) = 0.759, *p* = 0.484; *ηp*^2^ = 0.087) or treatment (F (1, 8) = 0.419, *p* = 0.536; *ηp*^2^ = 0.050) effects and no treatment × time interaction (F (2, 16) = 0.240, *p* = 0.790; *ηp*^2^ = 0.029) for normalized EMG (Table 1).

## 4. Discussion

Defined as “a psychobiological state caused by prolonged periods of demanding cognitive activity”, [9] mental fatigue is common in modern society as a result of occupations and leisure time that involve prolonged screen time [34,35,36,37,38]. Additionally, the specific stresses of daily life for athletes have been implicated as triggers of mental fatigue (reviewed by Schampheleer & Roelands [39]), including activities that are physically and cognitively demanding, such as endurance exercise and playing team sport [40,41,42]. Therefore, given the many potential causes, it is likely that mental fatigue may be present before and during training and competition. As mental fatigue may be associated with reduced motivation, commitment, attention and alertness, along with increased perceptions of effort, tiredness and distractibility [9], it may have significant implications for physical and cognitive performance during exercise and sport. Therefore, to better understand these effects, this study investigated whether experimentally induced mental fatigue, and associated changes in POMS, impacts maximal lower-body force production. Additionally, the effect of AIs on any mental-fatigue-related force loss was explored for the first time.

The AX-CPT used here induced mental fatigue, as evidenced by a marked increase in TMD caused by significant changes in the tension, depression, confusion, fatigue, anger and vigor POMS subscales [22,41]. In the present study, mental fatigue significantly reduced IMTP force immediately after participants completed the AX-CPT and second POMS questionnaire; however, the reduction in force was not significant when measured five minutes later. Resistance exercise has been shown to acutely improve mood states [43,44], and therefore it is possible that completing the first set of exercise post-AX-CPT positively altered participants’ moods so that later performance was no longer impacted. However, previous studies have used lower intensities, higher volumes and different timelines to assess changes in mood [44] and, as POMS was only measured after the AX-CPT and not after subsequent IMTPs or ammonia use, this is speculative and warrants further investigation. Alternatively, the effects of mental fatigue on strength may be transient, only lasting for a short period of time. Earlier studies have shown that mental fatigue can impair performance tasks that involve sustained attention or endurance [45]; however, its effects on brief, maximal muscular contraction may dissipate quickly due to the limited requirement for cognitive effort [9]. Either way, the effects of mental fatigue on strength are still relevant to time-sensitive competitive settings when mental fatigue may accumulate as a result of prolonged focus, repeated “psyching up” [1], emotional strain [3], and/or smartphone use [35,36,37].

Previous research investigating the effects of mental fatigue on force production has been inconsistent [13]; however, reductions in sustained submaximal and maximal isometric force have been reported. For example, a 9.4% decrease in knee extensor MVIC force was reported by Budini et al. [15] after 100 min of a repetitive computer task. Although no effect on maximal IMTP force was reported after 30 min of a modified Stroop test, Yang et al. [12] showed that mental fatigue negatively influences sustained force development. Similarly, strength endurance is affected by mental fatigue during dynamic upper and lower-body resistance exercise, with the number of repetitions to failure reduced after mentally fatiguing tasks of various durations [10,11]. The findings of this study support the theory that mental fatigue can be detrimental to strength performance, further supporting the need to control arousal before and during training and competition [1,2,37,46].

Reductions in IMTP force were not associated with changes in EMG, suggesting limited change in motor unit activation. Similarly, Alix-Fages et al. [46] reported that mental fatigue reduced muscular endurance and decreased motivation for their task, without altering neural drive. Further, Gantois et al. [37] suggest that the effects of mental fatigue on muscular performance are psychological in nature, increasing perceived effort and feelings of fatigue, as opposed to physiological alterations. Therefore, although motivation was not measured in the present study, changes in mood may have impacted participants’ motivation for performing the IMTP, with reduced vigor and elevations in negative emotions associated with reduced physical performance [47].

AIs did not mitigate the performance decline associated with mental fatigue. These findings align with previous studies that have failed to demonstrate a performance-enhancing effect of AIs on maximal dynamic and isometric strength tasks, despite causing an increase in psychophysiological arousal, feelings of being “psyched up” [20,48], and cerebral blood flow [32]. Relevant to the measures used in the present study, Perry et al. [32] and Bartolomei et al. [49] failed to observe a benefit of AI use on IMTP force, while no effect of AIs was seen during handgrip and knee extensor MVICs [20]. Equally, Vigil et al. [50] and Richmond et al. [51] reported no ergogenic effect of AIs on deadlift one repetition maximum (1 RM) and repetitions to failure with 85% 1 RM during the back squat, respectively. Although the effects of AIs on mental fatigue have not been investigated previously, Maleček et al. [52] found that AIs were unable to alter the effects of 36 h of total sleep deprivation; a condition that has been shown to reduce vigor and increase fatigue [53,54]. When taken together, the evidence strongly suggests that AIs offer no benefit to maximal strength, irrespective of mental state, and their continued use as an ergogenic aid is questionable. However, despite their obnoxious odor and effects on the respiratory system [19], the studies outlined above did not report any complications of AI use, suggesting that if an athlete believes that AIs provide a benefit as part of their pre-competition routine [4], then there may be no harm in using them. Athletes should do so with caution though, as AI use can cause severe acute reactions including rhinitis, conjunctivitis, dizziness, headache, shortness of breath and preorbital swelling when inhaled [55], and pharyngeal burns and vomiting if ingested accidentally [56].

Previous research has shown that inhaling pleasant or neutral odors, such as menthol, citral and green leaf smell (green odor), can alleviate the symptoms of mental fatigue [57,58,59]. Additionally, pleasant odors can improve mood, while unpleasant odors have the opposite effect [60]. The effects of odor on mental fatigue and mood are believed to be due to the relationships between the olfactory system and regions of the brain involved in mood, emotion, motivation, reward, and decision making [58,60,61]. Given the unpleasant odor of AIs, it is possible that they failed to alter, or further negatively impacted, mood, while also not impacting mental fatigue. However, as POMS was not measured after AI use, the effect of AIs on mood state is unclear and needs further investigation.

The major limitation of this study is the small sample size. Despite being powered to identify large changes in mood states after the AX-CPT, and finding a large, significant change in IMTP force over time (*p* = 0.009; *ηp*^2^ = 0.447), it is unclear whether the study was adequately powered to observe differences in force over time or between treatments when in a mentally fatigued state. As such, we cannot rule out the possibility of a type II error and, therefore, it may be prudent to repeat the study again using a larger sample size. Additionally, the study only investigated responses in resistance-trained males; therefore, future studies should include females, an underrepresented population in AI-related research despite showing positive responses to AI use prior to exercise [48]. A further limitation is the lack of a placebo, with only room air used as a control. Previous studies have used Vicks VapoRub™ [49,51], or water [20,48] as controls/placebos; however, given its strength and potent smell, it is impossible to blind participants to whether AI is being administered or not. Therefore, we cannot rule out the possibility of expectancy bias during AI trials as, despite being AI-naïve, participants may have expected some beneficial effect. Given the potential benefits of inhaling pleasant-smelling odors on the symptoms of mental fatigue [57,58,59], the use of an active control should be used in future research to compare the effects of pleasant and unpleasant smells on muscular performance in mentally fatigued and unfatigued states.

## 5. Conclusions

Mental fatigue increases total mood disturbance and, at the same time, reduces maximal lower-body isometric force. This finding has implications for those involved in sports/activities where mental fatigue may occur and maximal force production is required, such as during strength sport competition. Despite their popularity, this study suggests that AIs do not provide a benefit and should not be used to alleviate mental fatigue in strength sports. To better understand the effects of AIs on mental fatigue, and address this study’s limitations, future research should use a larger sample size, active placebo and include females.

## Figures and Tables

**Table 1:** Subscale scores for total mood disturbance (TMD) and profile of mood states before and following the AX-continuous performance test (AX-CPT) for the two treatment conditions (control and ammonia inhalation).

	Control				Ammonia Inhalation			
Subscale	Pre-AX-CPT	Post-AX-CPT	*p*	Cohen’s *d* [95% *CI*]	Pre-AX-CPT	Post-AX-CPT	*p*	Cohen’s *d* [95% *CI*]
Tension	10.62 ± 8.65	12.02 ± 6.84	0.336	0.18 [−1.13, 1.49]	9.78 ± 8.00	13.48 ± 8.45	0.006	0.45 [−0.87, 1.77]
Anger	7.89 ± 8.46	16.15 ± 10.68	0.099	0.86 [−0.51, 2.22]	7.67 ± 9.40	17.95 ± 10.94	0.028	1.01 [−0.38, 2.40]
Vigor	16.64 ± 3.72	8.30 ± 5.71	0.003	−1.73 [−3.26, −0.20]	16.47 ± 7.94	8.84 ± 4.86	0.004	−1.16 [−2.57, 0.25]
Depression	9.73 ± 10.47	14.10 ± 8.58	0.176	0.46 [−0.87, 1.78]	8.77 ± 9.49	15.90 ± 9.45	0.012	0.75 [−0.60, 2.11]
Confusion	8.14 ± 7.19	10.66 ± 6.08	0.126	0.38 [−0.94, 1.70]	6.38 ± 5.98	10.71 ± 6.70	0.005	0.68 [−0.66, 2.03]
Fatigue	11.63 ± 8.86	18.46 ± 6.17	0.012	0.90 [−0.48, 2.27]	6.61 ± 6.65	16.68 ± 5.24	0.002	1.68 [0.16, 3.20]
TMD	31.43 ± 42.40	63.04 ± 30.39	0.031	0.86 [−0.51, 2.22]	22.73 ± 35.37	65.88 ± 35.54	<0.001	1.22 [−0.21, 2.64]

Data are presented as the mean ± *SD*.

**Table 2:** Percent of maximum electromyography (EMG (%)), isometric midthigh pull (IMTP) force (N) and percent change in force (%Δ) at baseline, and immediately and 5 min after the AX-continuous performance test (AX-CPT) and second profile of mood states questionnaire for the two treatment conditions (control and ammonia inhalation). Significance (*p*) and effect sizes (Cohen’s *d* [95% *CI*]) are between baseline and immediately post AX-CPT, and baseline and 5 min post AX-CPT.

	Baseline	Immediately Post AX-CPT	*p*	Cohen’s *d* [95% *CI*]	5 min Post AX-CPT	*p*	Cohen’s *d* [95% *CI*]
Control							
EMG (%)	35.8 ± 9.0	33.6 ± 18.5	1.00	−0.15 [−1.46, 1.16]	34.2 ± 13.0	1.00	−0.14 [−1.45, 1.17]
IMTP (N)	1624 ± 301	1511 ± 342	0.482	−0.35 [−1.67, 0.97]	1523 ± 356	0.402	−0.31 [−1.62, 1.01]
IMTP (%Δ)	-	−6.6 ± 12.7	1.00	−0.74 [−2.09, 0.62]	−6.5 ± 10.0	1.00	−0.92 [−2.29, 0.46]
Ammonia Inhalation							
EMG (%)	39.5 ± 11.0	34.5 ± 10.9	0.206	−0.46 [−1.78, 0.87]	38.9 ± 18.4	1.00	−0.04 [−1.35, 1.27]
IMTP (N)	1774 ± 387	1531 ± 327	0.042	−0.69 [−2.02, 0.67]	1688 ± 365	0.707	−0.23 [−1.54, 1.08]
IMTP (%Δ)	-	−12.6 ± 12.3	0.859	−1.45 [−2.92, 0.02]	−4.1 ± 10.1	1.00	−0.57 [−1.91, 0.76]

Data are presented as the mean ± *SD*.

## Data Availability

The original data presented in the study are openly available in Figshare.com at https://doi.org/10.6084/m9.figshare.30369712.

## Abbreviations

The following abbreviations are used in this manuscript:

AI	Ammonia inhalant
AX-CPT	AX-continuous performance test
CON	Control
EMG	Electromyography
IMTP	Isometric midthigh pull
MVIC	Maximum voluntary contraction
POMS	Profile of mood states
TMD	Total mood disturbance
